# Avian Influenza: a global threat needing a global solution

**DOI:** 10.1186/1447-056X-7-5

**Published:** 2008-11-13

**Authors:** GCH Koh, TY Wong, SK Cheong, DSQ Koh

**Affiliations:** 1 Community, Occupational and Family Medicine Department, Yong Loo Lin School of Medicine, Singapore

## Abstract

There have been three influenza pandemics since the 1900s, of which the 1919–1919 flu pandemic had the highest mortality rates. The influenza virus infects both humans and birds, and mutates using two mechanisms: antigenic drift and antigenic shift. Currently, the H5N1 avian flu virus is limited to outbreaks among poultry and persons in direct contact to infected poultry, but the mortality rate among infected humans is high. Avian influenza (AI) is endemic in Asia as a result of unregulated poultry rearing in rural areas. Such birds often live in close proximity to humans and this increases the chance of genetic re-assortment between avian and human influenza viruses which may produce a mutant strain that is easily transmitted between humans. Once this happens, a global pandemic is likely. Unlike SARS, a person with influenza infection is contagious before the onset of case-defining symptoms which limits the effectiveness of case isolation as a control strategy. Researchers have shown that carefully orchestrated of public health measures could potentially limit the spread of an AI pandemic if implemented soon after the first cases appear. To successfully contain and control an AI pandemic, both national and global strategies are needed. National strategies include source surveillance and control, adequate stockpiles of anti-viral agents, timely production of flu vaccines and healthcare system readiness. Global strategies such as early integrated response, curbing the disease outbreak at source, utilization of global resources, continuing research and open communication are also critical.

## Background

Since the 1700s, there have been ten to thirteen influenza outbreaks or probable pandemics, of which three have occurred since the beginning of the 20^th ^century: the 1918–1919 Spanish flu pandemic, the 1957–1958 Asian flu pandemic and the 1968–1969 Hong Kong flu pandemic [1]. Of the three pandemics, the 1918–1919 pandemic was the most severe. The 1918–1919 strain of influenza was unusual because of the high rate of mortality among victims between the ages of 15 and 35 years. Deaths from influenza are usually due to secondary bacterial infection but many deaths during the 1918–1919 pandemic were caused directly by the virus itself. It appears that the immune system in young persons paradoxically went into over-drive while battling the influenza virus and progressed into an immunologic storm that killed the victims [2]. This was in contrast to the pandemics of 1957–1958 and 1968–1969 which were much milder. There were several reasons for this: the influenza strains were less virulent, the patterns of mortality were more typical of a usual seasonal influenza outbreak (i.e. it was concentrated among the very young and very old) and doctors were able to use antibiotics to treat secondary bacterial infections. The Attack Rate is the percentage of the population that becomes ill from an infection while case fatality rate refers to the percentage of infected people who die from the infection. Experts generally agree that the attack rates of the past 3 influenza outbreaks in the last century did not differ markedly and is estimated to be 25% to 30%. Using similar evidence, experts estimate the case fatality rate during the 1918 outbreak to be about 2.5% whereas the case fatality rates during the 1957–1958 and 1968–1969 episodes were below 0.2% [3].

The genes of the influenza virus can mutate in 2 main ways: (1) antigenic drift which involves small errors being incorporated into a virus gene sequence when the virus makes copies of itself and (2) antigenic shift involving an exchange of genes between two types of viruses (e.g. between avian and human forms of influenza virus) when both viruses are present in the same animal or human [4]. As a result of these mutations, the influenza virus changes its protein coat (antigens) and allows them to find new susceptible non-immune populations to infect. Both mechanisms of genetic mutation have the possibility of producing a new virus that can be easily transmitted between humans and initiate a pandemic. Scientists think that the 1918 influenza pandemic virus was a result of antigenic drift while the 1957–1958 and 1968–1969 influenza pandemic virus was a result of antigenic shift [5].

### Avian Influenza

The influenza virus has been in existence for centuries and has been constantly infecting both humans and animals (including birds). The *avian influenza *(AI) virus (also called *avian flu *or *bird flu *virus) is a subtype that causes contagious respiratory disease mainly in birds [6]. Wild waterfowls, especially ducks, are natural reservoirs and can carry the virus without manifesting symptoms of the disease and spread the virus over great distances. Domesticated poultry are also susceptible to avian flu and can cause varying symptoms ranging from reduced egg production to rapid death. The severe form of the disease is called "highly pathogenic avian influenza" (sometimes abbreviated as HPAI) and is associated with near 100% mortality rates among domesticated birds. AI has become endemic in several parts of Asia and it is believed that this is a result of unregulated poultry rearing practices in rural areas of developing countries. This is of concern because such birds often live in close proximity to humans and this increases the chance of genetic re-assortment between avian and human influenza viruses which may produce a mutant strain that is easily transmitted between humans [7,8].

In the past, avian influenza viruses have rarely caused severe disease in humans. However, in Hong Kong during 1997, a highly pathogenic strain of avian influenza of H5N1 subtype crossed from birds to humans who were in direct contact with diseased birds during an avian influenza outbreak among poultry. The cross-infection was confirmed by molecular studies which showed that the genetic makeup of the virus in humans were identical to those found in poultry. The H5N1 virus caused severe illness and high mortality among humans: among 18 persons who were infected, 6 died. The outbreak ended after authorities slaughtered Hong Kong's entire stock of 1.5 million poultry. Since then, AI among birds has been reported all over the world [9] and one of the factors responsible for the spread is the trans-oceanic and trans-continental migration of wild birds [10]. Most deaths from AI have occurred in Indonesia to date [11] and nearly all of the human cases resulted from close contact with infected birds [12]. However, there has been a reported cluster of plausible human-to-human transmission of the H5N1 virus within an extended family in the village of Kubu Sembelang in north Sumatra, Indonesia, in May 2006 [13].

Strains of influenza virus are classified into subtypes by their protein coat antigens, namely haemagglutin (HA) and neuramidase (NA). Of the 15 HA subtypes known, H1, H2 and H3 are known to have circulated among humans in the past century and hence, most people have gained immunity to interrupt the transmission of the virus. However, the H5N1 strain is unfamiliar to most humans and our low herd immunity to it poses a pandemic threat. There are thought to be three pre-requisites for a viral pandemic to occur: (1) the infectious strain is a new virus subtype which the population has little or no herd immunity; (2) the virus is able to replicate and cause serious illness and (3) the virus has the ability to be transmitted efficiently from human to human. The H5N1 virus satisfies the first two pre-requisites of a pandemic but has not developed the ability to be transmitted easily from human to human, yet.

### Lessons from the SARS Outbreak

The recent Severe Acute Respiratory Syndrome (SARS) virus outbreak in Asia saw another type of virus called the coronavirus spread widely in a short time. However, the SARS outbreak is considered to be "minor" when compared to the 1918–1919 influenza outbreak because less than 800 persons died from SARS worldwide whereas 40 to 50 million people died worldwide in the 1918 influenza pandemic [14]. However, the rapid spread of SARS to Asia, Australia, Europe and North America during the first two quarters of 2003 illustrates the speed that an AI pandemic can spread across the world. The major reason why SARS was quickly contained was that people with SARS were not contagious before the onset of case-defining symptoms which allowed effective control measures based on case-identification [15]. However, a person with influenza infection is contagious before the onset of case-defining symptoms, which limits the effectiveness of isolation of cases as a control strategy for this illness [16].

### The Feasibility of Early Containment Measures

The endemic nature of the avian flu among domestic birds and their close co-existence with humans in rural areas of Asia makes this part of world a likely epicenter of an AI pandemic. Two international teams of researchers used computer modeling to simulate what may happen if avian flu were to start being transmitted efficiently between people in Southeast Asia [17,18]. Both groups showed that a carefully selected and orchestrated combination of public health measures could potentially stop the spread of an avian flu pandemic if implemented soon after the first cases appear. Interventional strategies simulated include an international stockpile to 3 million courses of flu antiviral drugs, treating infected individuals and everyone in their social networks, closure of schools and workplaces, vaccinating (even with a low-efficacy vaccine) half the population before the start of a pandemic and quarantine measures. Targeted anti-viral treatment was a crucial component of all combined strategies and increasing public health measures needed to be greatly increased as the virus became more contagious. While the researchers said that implementing such a combination of approaches was challenging because it required a coordinated international response, the models did show that containing an avian flu pandemic at its source was theoretically feasible.

### Strategies to Contain and Cope with an Avian Flu Pandemic

To successfully contain and control an AI pandemic, both national and global strategies are needed [19]. National strategies need multi-pronged approaches and involve source surveillance and control, adequate stockpiles of anti-viral agents, timely production of flu vaccines and healthcare system readiness.

#### Source Surveillance and Control

When the H5N1 flu virus becomes easily transmissible from human to human, the earlier this fact is known, the more time there will be to gather and deploy available public health resources. Currently, the World Health Organisation (WHO), United Nations and other international agencies are trying to contain the H5N1 epidemic among poultry flocks in Asia and have set up monitoring systems to detect new outbreaks (especially human-to-human cases) early.

#### Flu Vaccines

Currently, there are ongoing efforts to mass produce and stockpile vaccines against the H5N1 strain. Recent models built on data from the 1918 flu pandemic predict that 50 million-80 million people could die and the overwhelming majority of deaths are likely to occur in the developing countries. Unfortunately, total global capacity for flu vaccine manufacture in the first 12 months is estimated at only 500 million doses. Moreover, flu vaccine production faces many constraints: the vaccine is cultured in eggs and this is a lengthy process which cannot be speeded up [20]. Fortunately, alternative sources of virus culture cells are being investigated. With avian flu affecting poultry and eggs, the egg supply required for vaccine production may itself be disrupted. Intellectual property rights and liability from adverse effects from vaccines are other issues that impede manufacturers from increasing vaccine production. It should also be noted that if the influenza pandemic strain turns out not to be the H5N1 variety, then the stockpiled vaccines would be useless and wasted. Deciding who to vaccinate is another challenge. Currently, influenza vaccination is recommended to the elderly and those with medical conditions which put them at higher risk for hospitalization and death if they become infected with influenza. However, some critics have argued that younger and healthier individuals should be given priority because they are more mobile than older, less healthy people and are therefore more likely to spread the flu to others. Another factor in favour of giving priority to younger people is that the seasonal flu vaccine produces a weaker immune response in the elderly. Moreover, if the flu pandemic has characteristics of the 1918–1919 pandemic, then the young and healthy are at higher risk of death. Even if supplies were adequate for all age groups, mass immunization for a potential pandemic still has its risks. In 1976, four US soldiers developed swine flu in an army camp and there was concern that it could become a pandemic like the 1918 Spanish flu. Although some health officials expressed doubts about the likelihood of an epidemic, the government initiated a mass inoculation programme for the entire US population. After hundreds of people receiving the vaccine came down with Guillian-Barre syndrome, the US government terminated the campaign and indemnified manufacturers, ultimately paying $93 million in claims [21].

There is a light at the end of the tunnel. The WHO recently announced plans to stockpile H5 influenza vaccine and create a policy framework for vaccine allocation and recommendations for its use [22]. Several recent developments in H5 vaccines have made this stockpile feasible: the development of H5N1 vaccines with adjuvants that reduce the required dose as much as fourfold [23] and the finding that adjuvant-enhanced vaccines may provide cross-protection against strains that have undergone up to seven years of genetic drift [24]. Furthermore, the manufacturing capacity of 500 million doses is calculated on a requirement for three strains of flu virus for standard vaccinations; in crisis mode, three times as much monovalent pandemic flu vaccine could be produced.

#### Antiviral Drugs

Anti-viral drugs are thought to be backbone of a management plan of an avian flu pandemic [25]. Only two anti-viral drugs have shown promise in treating avian influenza: oseltamivir (Tamiflu^®^) and zanamivir (Relenza^®^). A treatment of Tamiflu^® ^includes 10 pills taken over five days while Relenza^® ^is administered by oral inhalation. The US Food and Drug Administration has approved both anti-viral drugs for treating influenza but only Tamiflu^® ^has been approved to prevent influenza infection. Because antivirals can be stored without refrigeration and for longer periods than vaccines, developing a stockpile of antivirals has advantages as part of an overall strategy to control a flu epidemic. However, there are limitations to the use of antivirals: Tamiflu^® ^needs to be taken within 2 days of initial flu symptoms for it to be effective, but many people may not be aware that they have the flu early in the disease. Some research in animals and recent experience in the use of the drug to treat human cases have also found that Tamiflu may be less effective against the recent strains for the current H5N1 virus than the 1997 strain [26]. Improper compliance to antivirals by irresponsible individuals during an outbreak may results in the emergence of a drug-resistant strain. Lastly, there are current concerns about the safety of Tamiflu^® ^which has been associated with increased psychiatric symptoms among Japanese adolescents [27].

#### Healthcare System Readiness

Every country's healthcare system would be stretched to the limit in the event of a global pandemic of bird flu. The ability of healthcare facilities to maintain strict infection control measures would be challenged. The sudden surge in health manpower and facility need would be acutely felt among healthcare workers, epidemiologists and laboratory technicians. Countries must set up AI pandemic contingency plans and high-level coordinating committees comprising of representatives from multiple ministries and agencies.

#### International Strategies

An avian flu that is easily transmissible between humans would spread rapidly all over the world. The economic cost of an avian pandemic to all countries would be phenomenal and, if allowed to last for months, become exponential [28-30]. Early detection and control of an AI pandemic will also require a coordinated international response. Controlling avian flu is for the good of global public health and all countries have an interest and obligation to do so. Firstly, the response to the influenza threat would need an integrated cross-sector approach, bringing together animal and human health, areas of rural development and agriculture, economics, finance, planning and others. Partnerships are needed at both international and national levels. Next, there is certainly a priority on curbing the disease "at source" in the agricultural sector, thereby reducing the probability of a human epidemic. International resources are also needed for surveillance on avian influenza outbreaks and human-to-human transmission. It is also important to strike a balance between short and long term measures. Avian flu is becoming endemic in parts of East Asia and will require a long effort to suppress it. Meanwhile, a human pandemic may still emerge from a different strain of flu virus. Thus it makes sense for the international community to also undertake broader long-term measures to strengthen the institutional, regulatory and technical capacity of the animal health, human health and other relevant sectors in Asia. While country-level preparedness and leadership is essential for success, it must be backed by global resources. Even though the benefits of containing a pandemic are overwhelming, individual governments may still be daunted by the social, political and economic costs of various policy measures. Richer countries may have to support poorer countries in financial and non-financial means in the fight against a flu pandemic, for the sake of international good. The Global Outbreak Alert & Response Network (GOARN), a technical collaboration of existing institutions and networks who pool human and technical resources for the rapid identification, confirmation and response to disease outbreaks, is one such international body that supports global preparedness against bird flu. However, for such an organization to succeed, open communication and international cooperation is essential. Lastly, there is a critical need to share information rapidly with experts, policymakers and the worldwide community at large. Honest public communication will be critical as evidenced by China's denial of a local SARS outbreak initially which delayed early containment measures.

Recently, the Bill & Melinda Gates Foundation, the Pasteur Institute and the Wellcome Trust, began planning, with major medical-research funders and other stakeholders, several projects to enhance the research effort and reduce the risks from the threat of pandemic influenza over coming decades [22]. In the next few years, they plan to develop, maintain and disseminate a central inventory of funded research activities that are relevant to human influenza to ensure that stakeholders are well-informed. They will also coordinate road-mapping exercises to identify knowledge gaps to assist funders and researchers in establishing research-funding priorities, with specific focus on vaccines, drug therapies and epidemiology/population science (for example, diagnostics, surveillance, transmission and modelling), in the hope of developing a cohesive health-research agenda for pandemic influenza.

## Conclusion

In the words of the late Director General of World Health Organization, Dr Lee Jong Wook, '"it is only a matter of time before an avian flu virus acquires the ability to be transmitted from human to human, sparking the outbreak of human pandemic influenza...we don't know when this will happen but we do know that it will happen"[31]. Factors that suggest that an AI pandemic would be less severe than past influenza pandemics include advances in medicine such as the availability of antiviral medications and vaccines, and international surveillance systems. However, there are also factors that suggest than an avian influenza pandemic could be worse than the 1918 pandemic, such as a more densely populated world, a larger immunocompromised population of elderly and AIDS patients, and faster air travel and interconnections between countries and continents which will accelerate the spread of disease. Nevertheless, unlike the past, we have the prior knowledge of a possible impending pandemic and the knowledge of how to contain and control it. Preparedness, vigilance and cooperation, on local, national and international levels, are our best weapons against a deadly bird flu pandemic.

## Summary of Implications for GPs

Currently, the H5N1 avian flu virus is limited to outbreaks among poultry and persons in direct contact to infected poultry. Avian influenza (AI) is endemic in Asia where birds often live in close proximity to humans. This increases the chance of genetic re-assortment between avian and human influenza viruses which may produce a mutant strain that is easily transmitted between humans, resulting in a pandemic. Unlike SARS, a person with influenza infection is contagious before the onset of case-defining symptoms. Researchers have shown that carefully orchestrated of public health measures could potentially limit the spread of an AI pandemic if implemented soon after the first cases appear. Both national and international strategies are needed: National strategies include source surveillance and control, adequate anti-viral agents and vaccines, and healthcare system readiness; international strategies include early integrated response, curbing disease outbreak at source, utilization of global resources, continuing research and open communication.

## Authors' contributions

All authors contributed to development of the paper, writing of the manuscript and final approval for submission.
